# Revolutionizing the way students learn photographic arts through experiential education using AI and AR systems

**DOI:** 10.1038/s41598-025-24415-8

**Published:** 2025-11-19

**Authors:** Shashi Kant Gupta, Ahmed Alemran, Umi Salma Basha, Atiaf Ibrahim Zakari, SeongKi Kim, Raja Sarath Kumar Boddu, Sunil Kumar Vohra

**Affiliations:** 1 Adjunct Research Faculty, Centre for Research Impact & Outcome, Chitkara University Institute of Engineering and Technology, Chitkara University, Rajpura, 140401 Punjab India; 2 Department of Software Engineering, Medicine College, Misan University, Amarah, Iraq; 3 Computer Science and Engineering, Jazan University, Gizan, Saudi Arabia; 4 Computer Science & Engineering, Jazan University, Gizan, Saudi Arabia; 5 Chosun University, Gwangju, South Korea; 6 Raghu Engineering College, Visakhapatnam, India; 7 Institute for Career Studies, YMCA, New Delhi, India

**Keywords:** Augmented reality (AR), Smart classroom photography art, Deep recurrent neural network (DRNN), Artificial intelligence (AI), Education, Information systems and information technology, Mathematics and computing

## Abstract

The evolution of educational environments has seen a shift from conventional classrooms to technology-enhanced smart classrooms, driven by the rapid advancement of digital tools. The integration of traditional art education and modern technologies lacks interactivity and personalized feedback, which limits student engagement and creative progression. The objective of this research is to assess how AI and AR can be combined to improve student engagement, creativity, academic performance, and aesthetic understanding in art education. Data were collected from smart classroom sessions involving educational videos and interactive AR applications focused on photography. The pre-processing stage automatically filters low-quality images, retaining those with high saliency and clarity scores to ensure meaningful input for analysis. Using a TensorFlow-based experimental framework, a Deep Recurrent Neural Network (DRNN) algorithm was employed for intelligent image synthesis and feedback, allowing real-time analysis of composition and augmented visual storytelling. Results indicated notable improvements in student, Accuracy (97.18%), precision (97.33%), recall (96.95%), F1 score (97%). Students responded positively to the immersive experience, showing increased appreciation for cultural and visual diversity. In conclusion, the study demonstrates that integrating AI and AR in smart classroom environments can redefine art education by fostering experiential learning and providing dynamic, student-centered educational opportunities.

## Introduction

The concept of a classroom is evolving beyond four walls and a whiteboard. In today’s smart learning environments, intentionally integrated digital tools and technologies are embedded within the physical space to foster dynamic student interaction and participation on multiple social levels. These modern classrooms intend to amplify in-person communication while maintaining a shared base of knowledge between students and instructors. The hybrid space where technology supports, rather than replaces, human connection and collaborative learning^1^. Smart classrooms are founded on the pillars of innovative technologies that aim to improve teaching and learning. They include features such as facial recognition technology, video projectors, cameras, sensors, mobile learning, and environmental monitoring systems. These put together form a responsive and intelligent learning environment. It is not simply about digitizing conventional education but also about reengineering it into a more immersive, data-driven, and adaptive experience^2^. Artificial Intelligence (AI) is a key driver in smart classrooms, leveraging its potential to handle enormous datasets, identify patterns, and make data-driven choices. AI facilitates customized learning paths, instant feedback, and context-aware content delivery. AI-supported tools increase classroom interactivity and offer teachers insights that guide instructional planning. AI makes education more adaptive and responsive by ensuring that students access content that is attuned to their pace, interests, and learning difficulties^3^. Augmented Reality (AR), by contrast, superimposes digital information such as pictures, sounds, or 3D models over the actual surroundings. In the process, it enhances students’ sensory perceptions and enhances their conceptual grasp of abstractions. From being confined to gaming and entertainment only a few years ago, AR has now entered education, healthcare, design, and industry big time. Its visual-spatial interactivity is especially effective in arts disciplines like photography and visual arts^4^.

In photography art classes, the fusion of AI and AR is the start of a revolutionary period. The two technologies enable the integration of artistic creativity and technical advancements, transforming both the learning process and the role of the learner. AI enables picture analysis on autopilot, automatic editing recommendations, and pattern identification, whereas AR provides real-time visual augmentation so that students may create compositions prior to recording or include historical references in art projects. Together, they facilitate a more interactive, experiential, and engaging learning process, reshaping visual storytelling possibilities in the digital world^5^.

**Fig. 1:**
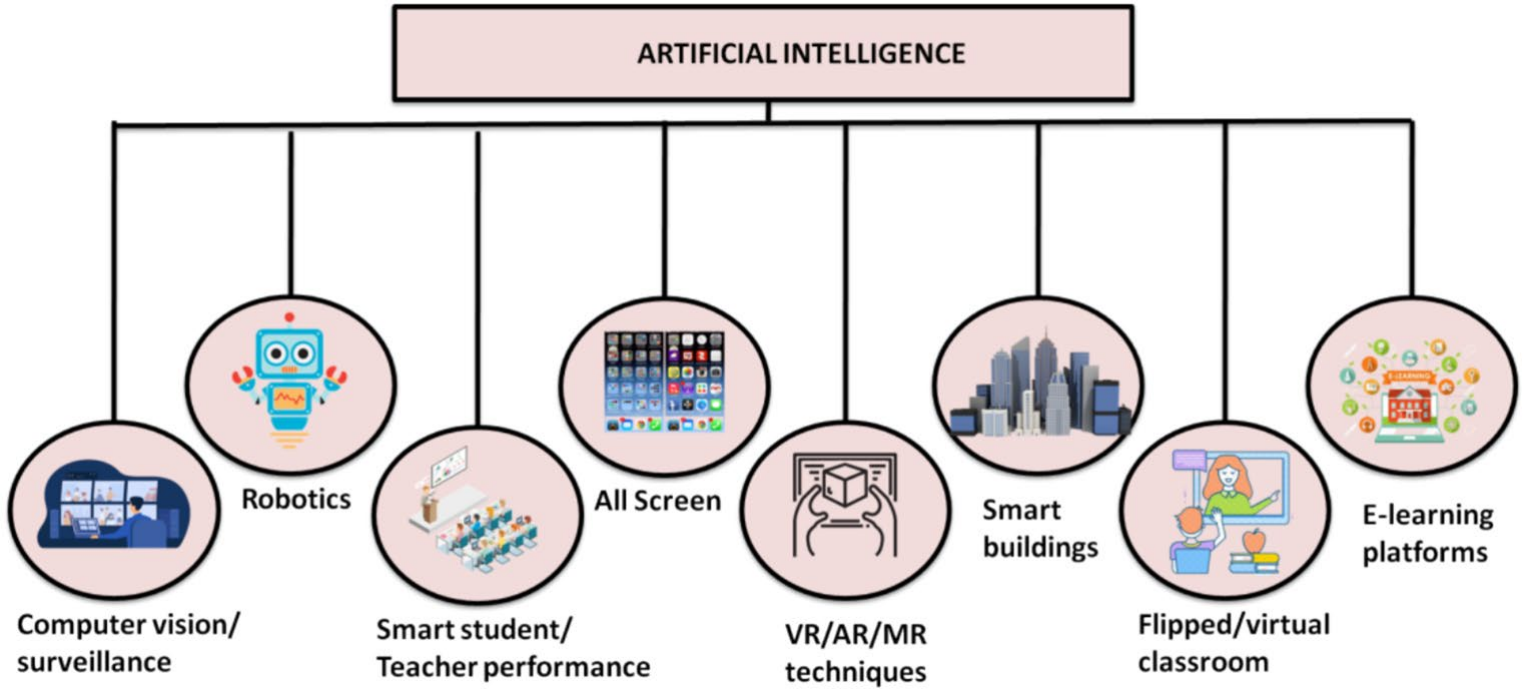
Technological tools used in a smart classroom.

A smart classroom can be either a real-world or online setting incorporating cutting-edge technology and AI to improve learning, as presented in Fig. 1.

Artificial Intelligence (AI) refers to the ability of computer systems to accomplish functions such as learning and reasoning, both functions that were typically related to human intelligence^6^. AI has come a long way since its inception, particularly with the invention of deep learning (DL), which has solved many machine learning (ML) problems and pushed the applicability of AI across domains. Within education, AI is not only focused on automating mundane teaching functions but also on driving intelligent systems, virtual assistants, and learning robots to enable personalized, interactive, and adaptive learning spaces^7^. AI has been widely applied across various educational technologies ranging from digital learning platforms and web-based chatbots to humanoid robots and intelligent tutoring systems^8^. While these technologies enhance educational delivery and access, few researchers have conducted comprehensive strengths, weaknesses, opportunities, and threats (SWOT) analysis or critically examined the full spectrum of the implications. Previous research includes smart classrooms and the underlying technologies. These include assessments of digital technologies, pedagogy, and models for adoption in technology-enabled learning environments^9^.

### Problem statement

In contrast to previous studies that provide wide-ranging statistical reports or superficial observations, this study targets the blending of AI and AR in photography art smart classrooms. It assesses how these technologies cooperate with teaching practices, learning processes, and artistic imagination^10^. This research is an attempt to find out how smart photo art classes with the integration of AI and AR increase student engagement, creativity, and performance by providing them with interactive experiences, smart image analysis, and experiential learning using state-of-the-art DL models such as DRNN and TensorFlow for the best possible educational achievements in visual arts.

### Research question

- How does the integration of AI and AR enhance student engagement, creativity, and academic performance in photographic art education within a smart classroom environment?
- Can the use of Deep Recurrent Neural Networks (DRNN) in combination with AR-based applications provide effective, real-time feedback and improve the aesthetic learning experience in visual arts education?

### Contribution of the research

- Innovative AI and AR Integration: Showcases the application of both AI and AR in tandem, particularly for teaching photographic art in smart schools.
- Saliency-based filtering is used in intelligent picture preprocessing to automatically choose high-quality images for better synthesis and analysis.
- The DRNN Framework for Art Learning uses a TensorFlow-based framework to apply a Deep Recurrent Neural Network (DRNN) for picture synthesis and real-time feedback, improving compositional comprehension.
- Student-Centered Evaluation: Offers factual proof that immersive learning enhances students’ creativity, engagement, visual literacy, and understanding of aesthetics.
- Engagement with Cultural and Visual Diversity: Emphasizes how interactive, inclusive, and experiential learning are supported by smart classrooms and foster an awareness of many creative viewpoints.

The rest of the research suggests literature review in "Related works" section. "Methodology" section contains the Analysis of Augmented reality for smart classroom photography art. "Result and discussion" and "Discussion" sections show the analysis of the result and discussion, and the research is concluded in "Conclusion" section.

## Related works

Research suggested architecture for the deployment of context-sensitive smart classrooms. It established a model that included technology integration, a context-aware prototype, and management practices^11^. The architecture enables adaptive learning environments through the ability to adapt to context changes. The research lacks empirical testing and real-world application, reducing generalizability and usability. To evaluate students’ readiness, interest, and attitudes in game-based learning settings in smart classrooms, a game-based learning strategy was put in place in an advanced learning environment, and students’ feedback was collected^12^.

Engagement increased, and students’ positive attitudes toward the approach were observed. Some limitations include the small sample size of participants and the limited time frame of assessment, which limits the generalizability of results. To examine the effectiveness of AI for BIM-enabled construction projects at a time of rapid digitalization, research conducted a bibliometric and content analysis of established designs and emerging trends^13^. Isolated dominant applications of AI across project life cycles and identified innovation trends. Lacked empirical validation and focused primarily on publications and in practice. Creating a Revolutionary Interactive Smart Classroom (RISC) used 5G for enhanced virtual learning^14^. Integration of haptic technologies, sensors, and 3D virtual services to replace human sensory input increased immersion and engagement in virtual classrooms. High cost, technical complexity, and dependence on the existing stable high-speed 5G infrastructure. To examine constantly changing definitions and frameworks for smart learning, highlighting flexibility, engagement, and personalized learning^15^. This literature-based editorial presents recent trends, pedagogical frameworks, and implementation techniques in smart learning. The research outlines contemporary practices in adaptive, collaborative, and intelligent learning. Theoretical perspectives are offered without scientific evidence or case studies on the efficacy of smart learning. The research examined the transition from traditional to digital learning and evaluated the impact of changes in technology on education^16^. Digital learning offers opportunities for flexibility, accessibility, and personalized experiences. Challenges include unequal access to technology, a lack of teacher training, and a lack of student engagement in virtual environments. To determine student learning styles in smart classrooms using Social Learning Analytics (SLA)^17^. External web and social network data used autonomous SLA cycles employed semantic mining, text mining, and data mining techniques. The SLA successfully generated dynamic knowledge models using semantic mining, enabling ongoing monitoring and improvements of ongoing learning processes. Practical application in the field was limited due to data privacy issues and the need for robust validation in wider educational contexts.

The aim was to determine student learning styles in smart classrooms using Social Learning Analytics (SLA)^18^. External web and social network data using autonomous SLA cycles employed Semantic Mining, text mining, and data mining techniques. The SLA successfully generated dynamic knowledge models using semantic mining, enabling ongoing monitoring and improvements of ongoing learning processes. Practical application in the field is limited due to data privacy issues and the need for robust validation in wider educational contexts. The research improved smart education through ML with a Hybrid 1D convolutional neural network long short-term memory (CNN-LSTM) architecture (edge, cloud)^19^. A hybrid architecture was implemented for analyzing multimodal data (text, images, video) from edge devices (e.g., smartphones, tablets, IoT) and from the cloud. This approach broadens the scope of personalization and awareness of student engagement and preferences in real time. However, computational intensity, data privacy, and reliance on reliable connectivity can be problematic. The research investigated the problems and new practices surrounding the incorporation of ML into K–12 computing^20^. Literature analysis and theoretical analysis of educational practices, frameworks, and paradigms related to ML in K–12, as introducing ML into K–12 computing required moving away from rule-based programming to data-driven thinking, and changing approaches to teaching computing. There was little empirical research on how K–12 students learn to develop and deploy ML systems, which leaves unfilled gaps in the research.

The objective was to recognize the key factors and challenges connected with the implementation of digital pathology (DP) in Italian pathology departments^21^. There was a series of discussions, including a first Zoom meeting and then a four-session in-person conversation that covered the definitions and applications of DP, the use of AI, and education. The successful implementation of DP requires automation, an appropriate and tailored scanner/microscope, and inter- and intra-disciplinary work. Barriers to the full success and implementation of DP include the high initial cost, regulatory gaps, and uncertainty in resolving questions regarding the storage of large volumes of virtual slides.

The research demonstrated the role of AI in education during the Covid-19 pandemic, as well as the development of a hybrid approach with Education Management Systems (EMS) with ML^22^. The current study was an experiential analysis of the online learning practices and AI applications throughout school lockdown and the use of AI as an enhanced delivery of online education to support both education and human learning. The hybrid approach should be tested to understand the scalability of the systems, the readiness of the infrastructure, and the duration of effectiveness.

Research assessed the application of Virtual Reality (VR) and AR within the classroom and their effectiveness for educational experiences^23^. The VR and AR technologies are compared to each other while using case studies of classroom applications as source information. VR and AR have the potential to improve student engagement, motivation, and learning results. Challenges include cost, access, and educators’ and institutions’ technological skills.

Research developed and assessed a smart workplace utilizing Internet of Things (IoT) capabilities to promote classroom efficiency and environment management^24^. Install temperature and light detection sensors; collect data through microcontroller and wired and wireless network debugging tools (backed up to dual-computer and cluster servers); achieve stable and prompt data transmission with 99.9% of the transmission under 30 ms; progress monitoring for smart campus development. High development costs were required, and substantial knowledge and expertise in design, implementation, use, upkeep, and maintenance.

Research evaluated AI methods employed to explore smart classroom data, improving teaching and learning^25^. Research included Systematic literature article, taxonomy creation, and aggregating AI uses in real educational practices. AI has potential in smart classrooms, but the majority of implementations are at low levels of readiness and lack consideration for educational impact. Real-world use was limited, and critical discussions of outcomes, such as data privacy and learning results, are lacking attention.

The limitations found across these studies presented key hurdles to the broad uptake of technology. As highlighted in the reviewed studies of VR and AR applications, high cost, limited access to VR devices, and insufficient technical knowledge of educators were obstacles to implementing solutions effectively^23^. In IoT-based smart classrooms, the cost of development and the need for specialized knowledge of system design, maintenance, and operation all posed challenges^24^. It was evident that AI-based approaches hold promise, but they also mostly remain in development stages and have not been deployed in the real world and have little real-world deployment. In addition, many associated issues were not considered, such as privacy, educational impacts, or determining learning outcomes^25^. The present research overcomes these limitations by applying AI and AR in a cost-effective and scalable framework such as TensorFlow, thereby limiting the need for hardware. It considers associated issues such as training educators, privacy of data, and user-friendly platforms with real-time feedback systems to increase usability. Concentrating on potential applications in art education, it allows for meaningful, immersive learning experiences and measurable academic achievements and creative outcomes.

The ethical implications of using ChatGPT in ASEAN higher education systems are examined in the research^26^. Key ethical issues related to data privacy, algorithmic bias, and the influence on educational practices are highlighted by the research using a comprehensive literature review and thematic analysis of data from news items, university policies, and journal papers. From a socio-technical perspective, the findings emphasize the need for responsible generative AI usage and offer recommendations for ensuring that ChatGPT and related AI technologies support academic integrity and equity while making a positive contribution to higher education.

The value and potential hazards of metaverse-based mental healthcare applications are examined in the research^27^. An efficacy assessment of four digital therapy applications NightWare, Freespira, EndeavorRx, and Sleepio was performed on disorders of posttraumatic stress disorder (PTSD), anxiety, and attention deficit hyperactivity disorder (ADHD) by means of systematic literature review (SLR) and case study analysis. The results indicate that metaverse-based treatments provide considerable potential benefits with clinical validation for efficacy. Long-term efficacy, accessibility and privacy of use remain limitations.

The research analyzed the Shwedagon Pagoda in Myanmar, Angkor Wat in Cambodia, and Kodaiji Temple in Japan are identified as three temples of cultural significance in Asia that are researched using case study design^28^. The three temples are selected in the study to illustrate and compare the varying digital preservation methods in the metaverse based on religious, historical, and architectural merit. Kodaiji Temple, a Zen Buddhist shrine in Japan, uniquely illustrates how VR can depict not only architectural beauty but also an atmosphere of spirituality. There are limitations to this research, especially related to cultural sensitivities as they apply to digitizing sacred spaces, as well as access to high-quality high-resolution VR models.

Exploring the effects of immersive technologies and how they can affect both educators’ and students’ academic experiences is the aim of the research^29^. In academic innovations and interdisciplinary research, it aims to provide light on the possible trends of immersive technology, including the metaverse, in both current and future educational institutions. The impact of immersive technology and advanced digital learning on the current educational systems is examined using a methodical research approach.

## Methodology

**Fig. 2:**
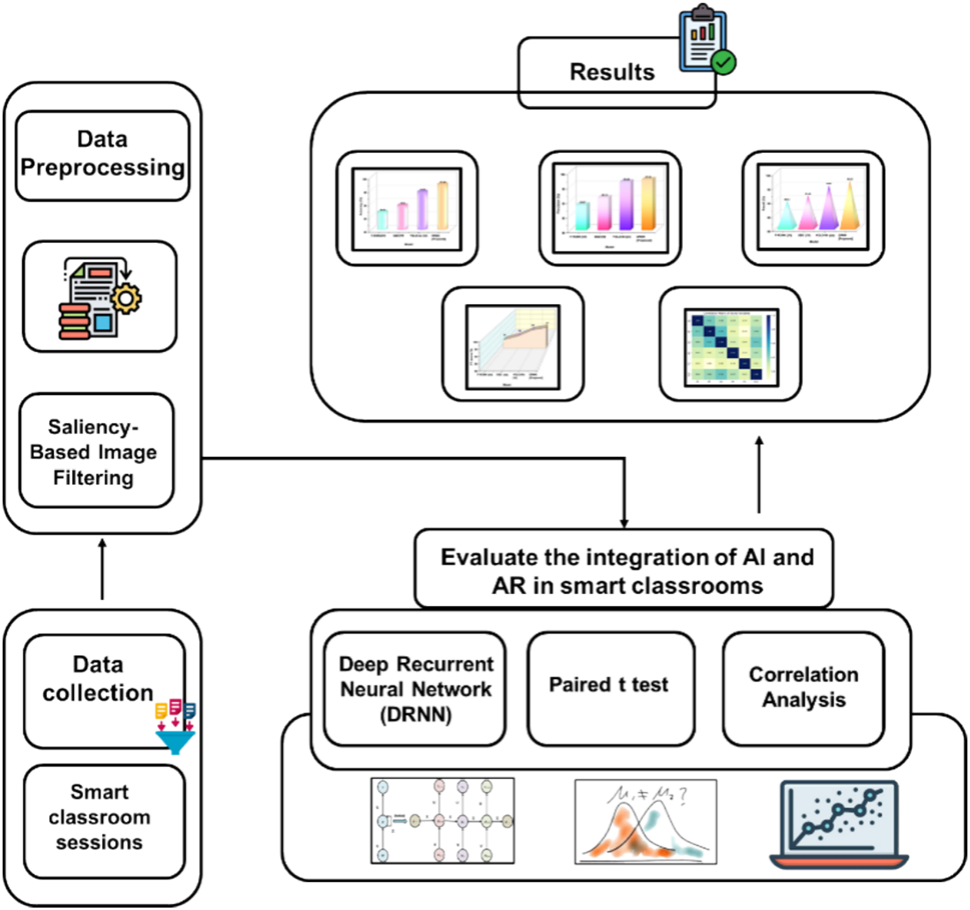
Proposed flow of AI-AR Smart Classroom Learning Enhancement.

This research utilized an integration of AI and AR in a smart photography classroom. Data was collected through the use of educational videos that showed the process taken to create the photographs, on which students were assessed through performance measures, surveys, and observation of their performance. Data Processing utilized saliency-based filtering to improve the image quality prior to applying a DRNN model using TensorFlow. The AR apps support interactive learning and allow it to happen. This method increased student engagement, augmented creativity and image analysis skills, and showed promise in creating improvements in educational outcomes within the field of photography and art. Figure 2 shows the workflow illustrating the implementation of AI and AR techniques for enhancing learning in smart classroom environments.

### Basic for augmented reality (AR)

AR is frequently used in smart classrooms to provide students with more engaging educational opportunities. By superimposing computer-generated imagery (CGI) onto a user’s real-world surroundings, AR provides consumers with a novel and engaging way to interact with content. Tablets, smartphones, smartboards, and a variety of applications all fall under the category of smart class augmented display devices.

#### Designing videos for augmented reality instruction

**Fig. 3:**
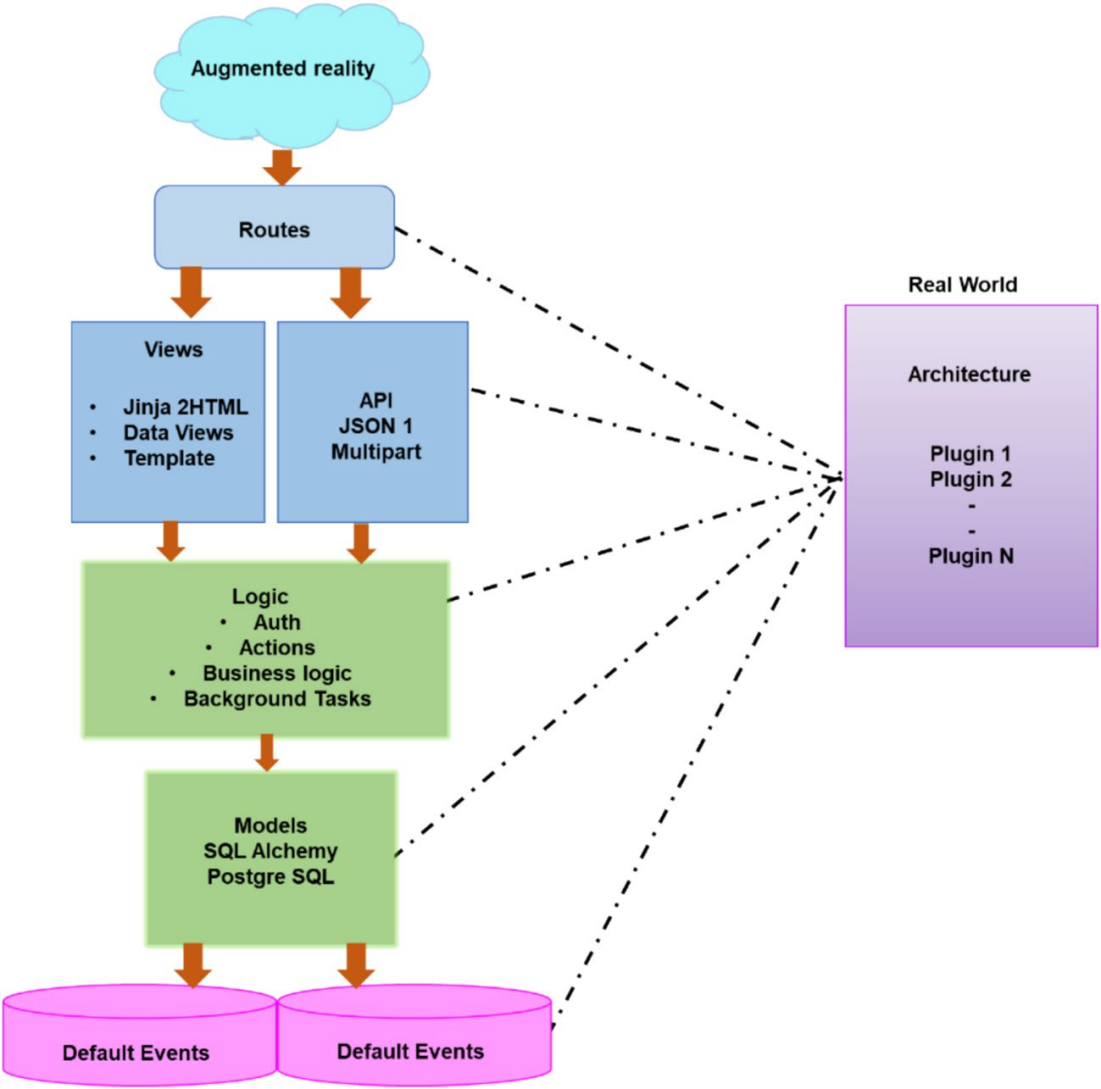
AR architecture.

AR incorporates a wide range of technological components from several domains and disciplines as a holistic technology. The three-dimensional, computer-generated virtual world or space produced by the system simulation exists inside the computer. Through engagement, people may experience a virtual world or environment because of the realistic sensory input it provides, giving them the impression they are in person. According to some academics, AR is a type of advanced human–computer interface that enables individuals to engage with their surroundings and communicate effectively in a three-dimensional virtual world. Figure 3 illustrates a specific partition of the functional needs of AR devices into three systems based on this defined division.

For engagement, feedback, and real-time information sharing, the user helped in perfectly simulating a three-dimensional reality through the measurement system for the behavior of action and operation, perception, and computation. Data simulation is used to develop a simulation system through a representation system to provide commands for actions and behaviors, User engagement, information transfer to the user, and feedback for the user.

### Data collection

Smart classroom learning data were collected from open source kaggle. This dataset records 1020 rows of learning results, student innovation, and engagement in an intelligent classroom setting. Performance measures including visual literacy, aesthetic comprehension, and engagement with augmented reality applications during photography art workshops are reflected in the data. Saliency scores, session lengths, and final grades are all included.

Source: (https://www.kaggle.com/datasets/programmer3/smartclassroom-ai-ar-photography-insights).

### Data preprocessing using saliency-based image filtering

Preprocessing is the first step in data analysis, where raw data is transformed into a cleaned, usable format for subsequent processing. Saliency-Based Image Filtering is a preprocessing mechanism focused on retaining notions of visual significance through analyses of features such as contrast, color, and spatial location within the image. Equation (1) shows that the filtering step allows to improving critical details while reducing noise and computation through visual learning tasks.

1 $$\begin{aligned} {V}_{j} & =\sum_{i=1}^{M}\Vert {d}_{j}-{d}_{i}\Vert { }^{2}{x}_{ji}^{\left(u\right)}\\ & ={D}_{j}^{2}\sum_{i=1}^{M}{x}_{ji}^{(u)}-{D}_{j}^{2}\sum_{i=1}^{M}{{d}_{i}x}_{ji}^{(u)}+\sum_{i=1}^{M}{{d}_{i}^{2}x}_{ji}^{(u)} \end{aligned}$$

In this equation, we are calculating the informed weighted variance ${V}_{j}$. It is based on the distances between the pixel values ${d}_{j}-{d}_{i}$ and saliency weights ${x}_{ji}^{\left(u\right)}$, which represent visual significance. In the preprocessing component of research, this method filters out non-fruitful image data by identifying salient regions. Through this method, AI-AR integration accordingly elevates the perception of meaningful composition in the analysis of learning to photograph in a smart classroom.

### Deep recurrent neural network (DRNN)

**Fig. 4:**
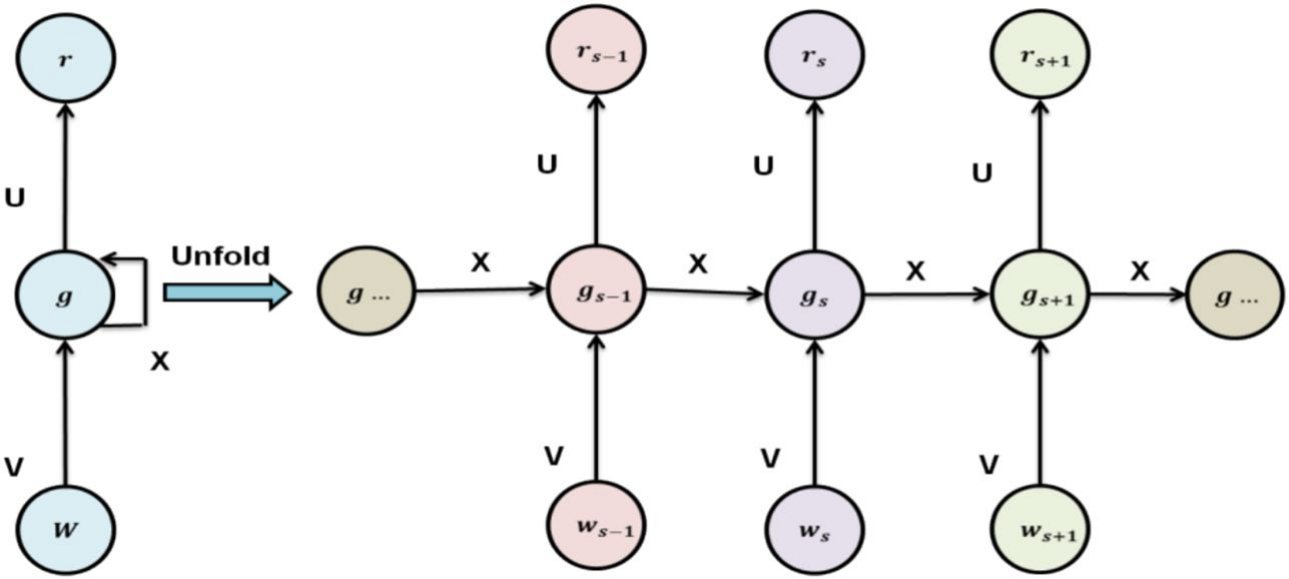
Structure of DRNN.

Deep Recurrent Neural Network (DRNN) algorithm was employed for intelligent image synthesis and feedback, allowing real-time analysis of composition and augmented visual storytelling. DRNN is exceptionally well-suited because of its effectiveness in dealing with time series issues. The DRNN is effectively used for parameter projection in many applications, including forecasting, image processing, and many industries. The output response is assessed using a cycle of feedback that includes the concealed output from the current and the previous instance’s buried output. In the feedback loop of the previous phase, the data is recorded, and the final production is projected using both the immediate and intermediate outputs of the prior step. Figure 4 displays the DRNN’s fundamental structure.

The computation is performed using the DRNN algorithm at time t with input series $(w = w1 \dots {w}_{j})$, hidden vector series $(g = g1, \dots {g}_{j})$, and output vector *yk*. The series’ mathematical expressions are displayed below. Equations (2–5) show forward propagation equations that use input characteristics, recurrent connections, and bias factors to calculate activations in the DRNN.

2 $${net}_{i}=\sum_{i}{x}_{j,i}{w}_{j}+{x}_{gg}{g}_{j-1}+{\theta }_{j,i}$$

3 $${P}_{i}=e\left({net}_{i}\right)$$

4 $${net}_{l}=\sum_{l}{X}_{j,i}{P}_{i}+{\theta }_{i,l}$$

5 $${P}_{l}=e\left({net}_{l}\right)$$

where ${net}_{i}$ indicates the weighted average of the input layer and the hidden layer, ${x}_{j,i}{w}_{j}$ indicates the weight of a buried layer ${P}_{i}$ concerning subsequent time steps, and $e\left({net}_{i}\right)$ is the ratio of the concealed layer’s importance to the output layers. The ${\theta }_{i,l}$ display the results of the hidden and output layers, respectively. Equation (6) illustrates a sigmoid activation function used to determine output probabilities in the DRNN model.

6 $$e\left(net\right)=\frac{1}{{1+e}^{(-net)}}$$

Real-time recurrent learning (RTRL) and back-propagation through time (BPTT) are two training techniques that may be employed to train the DRNN. Specifically, BPTT switches the network parameter from feedback to feed-forward architectures. The BPTT approach, which consists of two main stages, the forward pass and backward pass, is used for this research. The backward pass technique, in contrast, calculates the mistake and transmits data from the output layer to the hidden layer. The mathematical equation below provides an approximation of the output layer error. Equation (7) shows the error function that shows the discrepancy between the DRNN model’s expected output and actual output.

7 $${f}_{l}={S}_{l}-{P}_{l}$$

where, DRNN-based smart classroom system, ${S}_{l}$ represents the error function where ${S}_{l}$ is the ground truth saliency score and ${P}_{l}$ is the prediction. This error is used to tell the network how to improve its image-based artistic feedback for photography pedagogy. Equation (8), the derivative of the activation function in DRNN, is used to calculate the gradient of the loss concerning the output layer net input.

8 $${\partial }_{l}={f}_{l}{\prime}e({net}_{l})$$

The DRNN-based model smart classroom incorporates photographic art. The equation partial ${\partial }_{l}$ defines local gradient as the derivative of an activation function ${f}_{l}$ multiplied by the error at layer ${f}_{l}{\prime}e({net}_{l})$. This is used to propagate backwards in a timely and intelligent manner for producing images and feedback. In Eq. (9), the DRNN training process, the gradient of the hidden layer node is calculated using the backpropagated error from the output layer and the derivative of its stimulation.

9 $${\partial }_{i}=e{\prime}({net}_{i}){\partial }_{l}{x}_{i,l}$$

The equation ${net}_{i}$ computes the backpropagated error at neuron $i$ in the context of DRNN-driven smart classroom $e{\prime}({net}_{i})$ for photographic art, where ${\partial }_{l}{x}_{i,l}$ is the connection weight. This aids in optimizing the model to provide individualized creative feedback and image-based learning. Equations (10–12) show that learning rate, error gradients, and prior activations are used in the DRNN model’s weight and gate update equations to maximize learning in smart classroom picture synthesis.

10 $${\Delta x}_{i,l}=\alpha {\partial }_{l}{P}_{i}$$

11 $${x}_{i,l}={\Delta x}_{i,l}+{\Delta x}_{i,l}$$

12 $${\Delta x}_{gg}=\alpha {\partial }_{i}{g}_{j-1}$$

The weight update method during training is described by the equations employing DRNN for smart classroom photographic art. Based on the learning rate, ${\Delta x}_{i,l}$ modifies the weight. Weights are further refined by ${\Delta x}_{gg}$ and error gradient $\alpha {\partial }_{i}{g}_{j-1}$ to enhance visual learning predictions and tailored feedback. Equations (13–15) maximize feature learning in smart classroom settings, update the DRNN’s rules for recurrent connections and input weights by using gradient-based modifications.

13 $${x}_{gg}={x}_{gg}+{\Delta x}_{gg}$$

14 $${\Delta x}_{j,i}=\alpha {\partial }_{i}{w}_{j}$$

15 $${x}_{j,i}={x}_{j,i}+{\Delta x}_{j,i}$$

The repeated weight updates in these equations are consistent, which is intended to improve photographic art instruction using DRNN. While ${x}_{gg}+{\Delta x}_{gg}$ modifies inter-layer weights, the expression $\alpha {\partial }_{i}{w}_{j}$ updates recurrent weights using gradients. Equations (16–19) show the modifications that maximize the network’s learning precision for instructional materials that rely on images.

16 $${\Delta \theta }_{i,l}=\alpha {\partial }_{l}$$

17 $${\theta }_{i,l}={\theta }_{i,l}+{\Delta \theta }_{i,l}$$

18 $${\Delta \theta }_{j,i}=\alpha {\partial }_{i}$$

19 $${\theta }_{j,i}={\theta }_{j,i}+{\Delta \theta }_{j,i}$$

The use of the formula ${\Delta \theta }_{i,l}$. The gradient-based updates of weights across layers are represented by the equation ${\Delta \theta }_{j, i}$. This facilitates the model’s efficient learning of artistic traits from preprocessed picture data. The DRNN based on the BPTT algorithm is shown in Algorithm 1.

**Algorithm 1:**
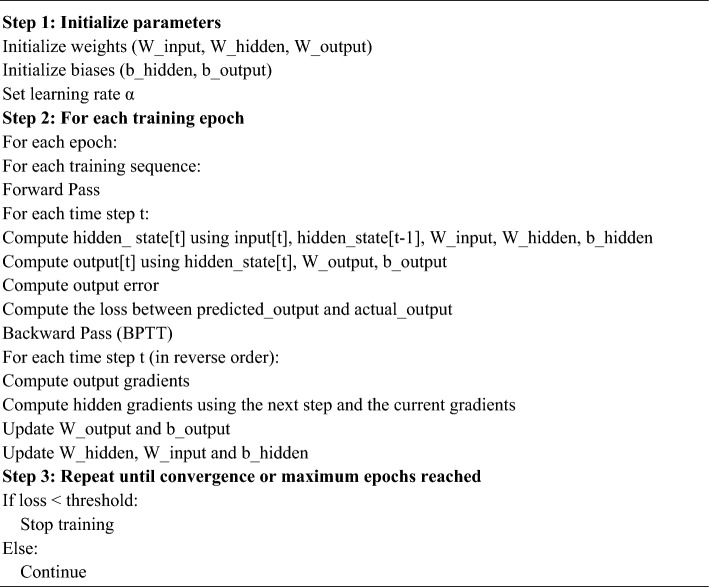
DRNN algorithm.

### Parameter setup

The hyperparameter for DRNN method as shown in Table 1

**Table 1:** Parameters setup.

Hyperparameters	Typical Values
Hidden units per dense layer	128, 256, 512
Epochs	50, 100
Dropout rate	0.3, 0.5, 0.6
Optimizer	Adam, RMSProp
Learning rate	0.001, 0.0001
Number of recurrent layers	2, 3
Activation function	Sigmoid, Tanh
Sequence length	10, 20
BPTT Truncation steps	5, 10

## Result and discussion

The goal was to improve learning outcomes, creativity, and student engagement, in the teaching of photographic art by incorporating AR and AI into a smart classroom setting. This section discusses the findings of the model’s use, including comparative analysis and performance evaluation.

### Comparison phase

The proposed DRNN model was compared with existing computer vision-based methods, including Faster Region-Based Convolutional Neural Network (F-RCNN)^30^, Single Shot Detector (SSD)^30^, and You Only Look Once version 5 nano (YOLOv5n)^30^. These models are widely used for object detection tasks and have demonstrated strong performance in general image analysis.

**Table:** 

Model	Accuracy (%)	Precision (%)	Recall (%)	F1 score (%)
F-RCNN^30^	86.68	88.67	89.63	87
SSD^30^	89.2	91.15	91.66	90
YOLOv5n^30^	94.32	96.54	95.41	95
DRNN [proposed]	97.18	97.33	96.95	97

*Accuracy:* The term accuracy refers to the proportion of correctly identified outcomes, e.g., the elements relevant to photography or patterns of learning by the DRNN model as it informs outcomes in smart classroom sessions. Equation (20) indicates the degree to which the intelligent agent is adept at recognizing and acting upon meaningful content. As accuracy goes up, it suggests a greater level of AI and AR integration to support outcomes in art education.

20 $$Accuracy=\frac{TP+TN}{TP+TN+FP+FN}$$

**Fig. 5:**
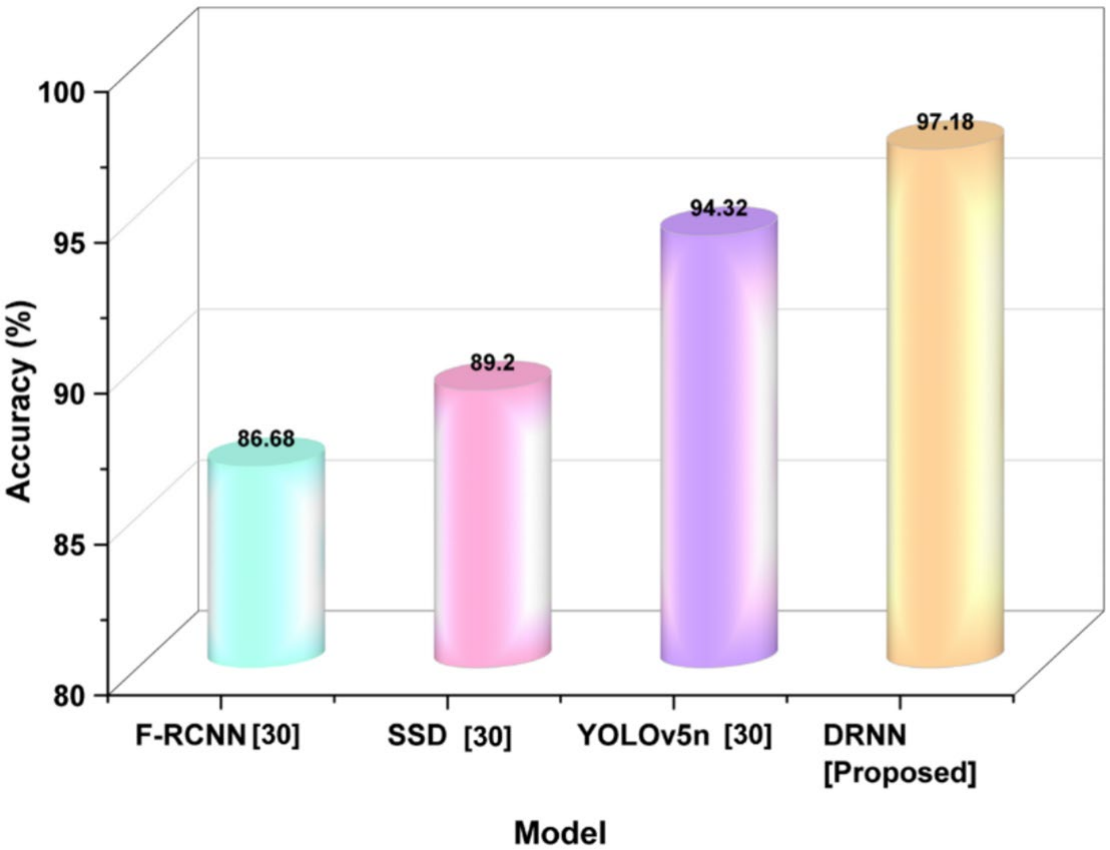
Accuracy outcome across selected models.

By increasing photographic art education using AI and AR, accuracy indicates the model’s precision in recognizing the relevant learning outcomes. In this case, TP and TN are accurate predictions, while FP and FN indicate unreliable evaluations in the student’s input or image relevance. Figure 5 shows a model accuracy comparison: DRNN achieves the highest overall prediction correctness.

The DRNN model produced the highest accuracy of 97.18%, outperforming YOLOv5n (94.32%), SSD (89.2%), and F-RCNN (86.68%). Accuracy shows how correct the predictions of the model are. It is the fraction of right predictions (true positives and true negatives) among all predictions.

*Precision:* The term precision specifies the DRNN model performing the identification and classification of quality, salient images that will be used for formal analysis. It specifies the extent to which images that are labeled as relevant help optimize creative and aesthetic learning, which was evaluated using Eq. (21). High precision denotes whether feedback from the intelligent agent was characterized as effective and purposeful in smart classroom environments.

21 $$Precision=\frac{TP}{TP+FP}$$

**Fig. 6:**
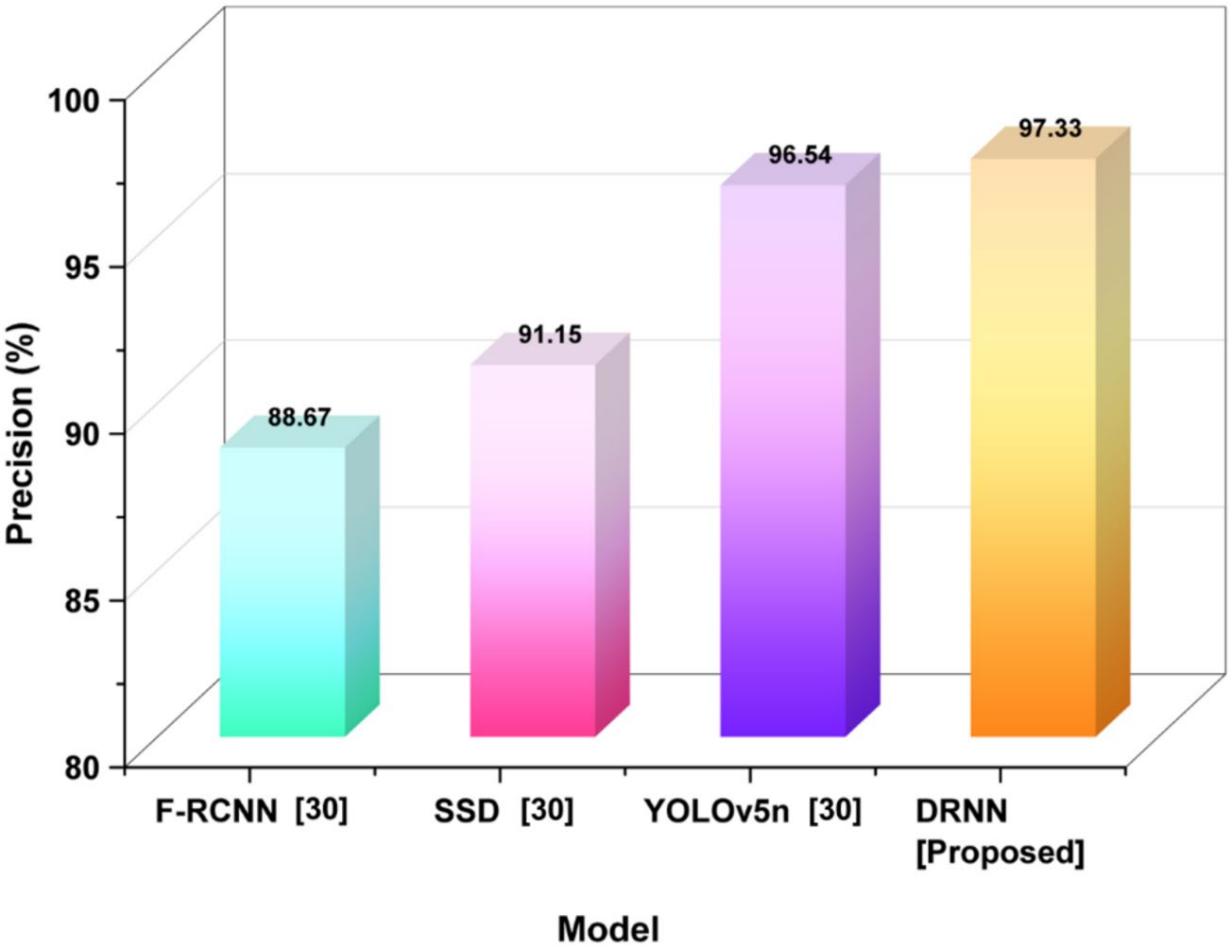
Precision results reflecting model classification quality.

Precision measures how accurately the AI model detects relevant artistic features from student-created images. The AI system identifies TP quality images and FP as low-quality images that the AI system considers acceptable. Figure 6 shows precision rates across models: DRNN shows superior accuracy in identifying true positives.

Precision indicates the number of positively predicted cases that were truly correct. DRNN’s precise percent was 97.33%, which is higher than both YOLOv5n (96.54%), SSD (91.15%), and F-RCNN (88.67%), meaning it has a better ability to minimize false positives.

*Recall:* The term recall measures the capability of the DRNN model to capture correct image features that are relevant and of high quality in the smart classroom environment. Recall is pertinent in making sure the model is not missing any vital elements in determining any significant visual details as it analyzes and creates photographic outcomes evaluated using Eq. (22).

22 $$Recall=\frac{TP}{TP+FN}$$

**Fig. 7:**
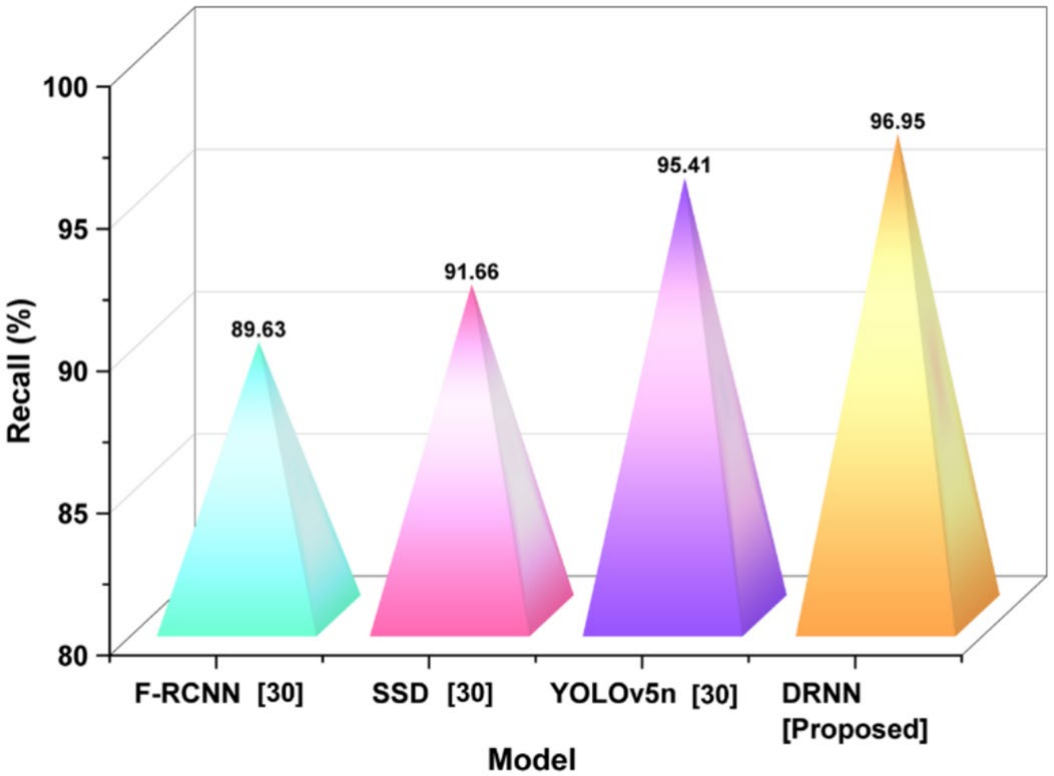
Recall performance for detection consistency.

The AI-AR system does in identifying relevant, student-generated visual content for feedback. To clarify, TP refers to the identification of high-quality images. FN refers to missed examples flagged for consideration and improvement, as shown in Fig. 7.

Recall indicates the model’s ability to find all relevant instances. DRNN’s recall was 96.95%, indicating a strong ability to detect true positives, higher than YOLOv5n (95.41%), SSD (91.66%), and F-RCNN (89.63%).

*F1 Score:* The metric F1 score is another performance metric used to assess if the DRNN model is indeed intelligently producing correct photographic opportunities, as it represents the harmonic mean of precision and recall, making sure that the balance between the identification of artistic elements is not hampered by falsely classified images. Equation (23) shows that the performance illustrates the role of AI as somewhat of a validator for visual learning outcomes.

23 $$F1-score=\frac{Precision\times Recall}{Precision+Recall}$$

**Fig. 8:**
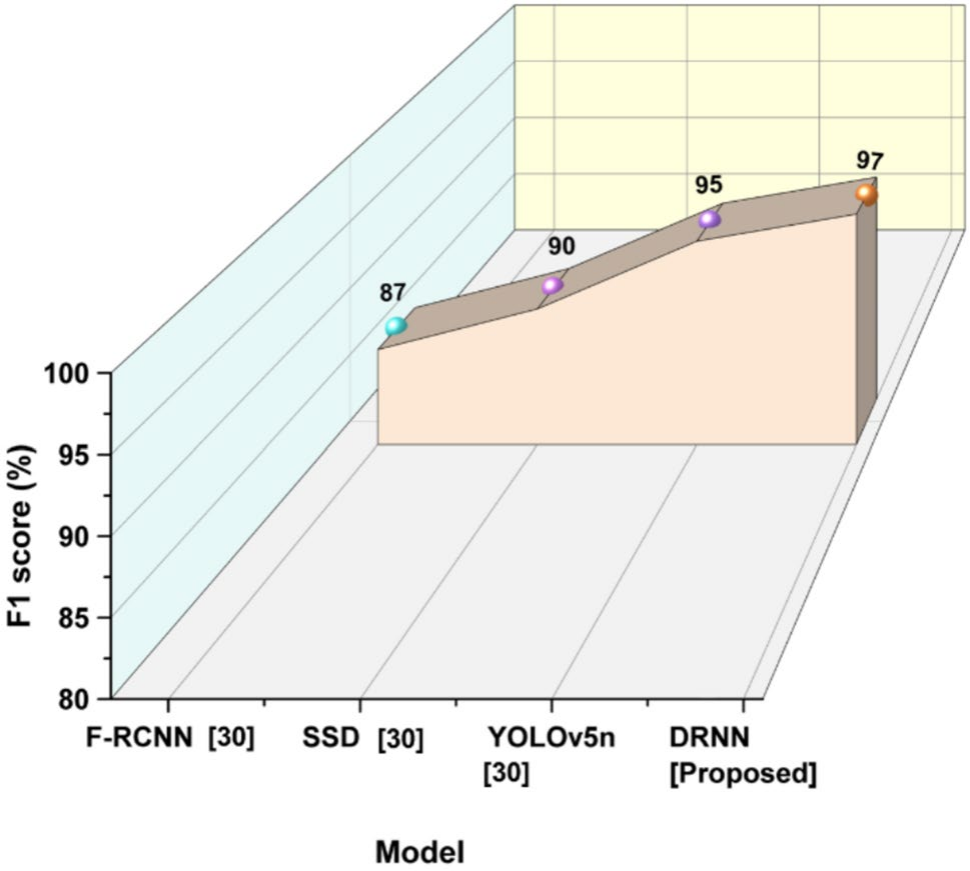
F1-score outcome indicating overall model effectiveness.

Figure 8 shows that the F1-score provides effective feedback and content analysis of photographic art education.

The F1 score is a measurement of pure model performance that balances precision and recall. DRNN had the highest F1 score of 97% in four model comparisons, whereas YOLOv5n (95%), SSD (90%), and F-RCNN (87%) were behind. In summary, DRNN has strong and balanced detection capabilities.

### Pilot study

A pilot study is a small-scale exploratory examination that is carried out to assess a planned research project’s viability, duration, cost, and possible impact. Before the main study, it assists with identifying and resolving methodological difficulties. This study evaluated the efficacy of integrating AI and AR in art teaching through a pilot study involving thirty students.

#### Participants profile

The pilot study consisted of 30 participants with an equal gender ratio, and most of them ranged in age from 18 to 26 years. Most participants had some experience in Visual Arts, while two-thirds had never experienced AR before, and most said they had only a basic familiarity with AI, indicating the need for some buy-in and basing support around emerging technologies, as shown in Table 2.

**Table 2:** Demographic distribution of participants.

Variables	Category	Frequency (n)	Percentage (%)
Gender	Male	15	50.0
	Female	15	50.0
Age group	18–20 years	12	40.0
	21–23 years	13	43.3
	24–26 years	5	16.7
Educational background	Visual Arts	18	60.0
	Multimedia/design	7	23.3
	Other (e.g., CS, education)	5	16.7
Prior AR experience	Yes	10	33.3
	No	20	66.7
Familiarity with AI	Basic	21	70.0
	Intermediate	6	20.0
	Advanced	3	10.0

#### Variables explanation

- *Student engagement (SE):* Assesses the degree to which students are paying attention, showing interest, and actively participating in AI and AR-enhanced learning activities. Higher levels of engagement are usually linked to better learning outcomes and motivation.
- *Academic performance (AP):* Refers to students’ success on applied learning assessments and other assigned tasks. It serves as an evaluative technique that is used to determine the extent to which students have academically learned in the context of embedding AI and AR.
- *Creative output (CO):* Evaluates students’ originality and inventive possibilities in photographic art tasks. This variable demonstrates how immersive tools provoke artistic meaning-making.
- *Aesthetic appreciation (AA):* Articulates students’ ability to identify, analyze, and appreciate artistic and visual qualities. It iss evidence of increased cultural and artistic awareness.
- *Technology usability (TU):* This measures how students and educators utilize AI and AR tools in the smart classroom. Good usability leads to more seamless learning experiences.
- *Visual literacy development (VLD):* The variable measures students’ ability to assess, interpret, and create visual outputs. This is an important skill in photography education, especially through the lens of AR and AI.

## Paired t-test

A statistical technique for comparing the means of two related groups to see if there is a significant difference between them is the paired t-test shown in Eq. (24). It is frequently used in educational research to measure the same participants’ pre- and post-test scores before and after an intervention.

24 $$s=\frac{\sum c}{\sqrt{\frac{m\left({c}^{2}\right)-{\left(\sum c\right)}^{2}}{m-1}}}$$

This equation represents the t-statistic formula for paired t-tests, with $s$ measuring the standardized mean difference. Here, sum $c$ represents the sum of paired differences, $m$ is the number of pairs, and the denominator calculates the standard error of the mean difference (Table 3).

**Table 3:** Outcome of paired T-test analysis highlighting statistically significant enhancements.

Variable	Mean (Pre)	Mean (Post)	Mean difference	t-value	df	p-value	Significance
SE	3.12	4.26	1.14	6.72	29	< 0.001	Significant
AP	68.4	79.2	10.8	5.89	29	< 0.001	Significant
CO	2.87	4.03	1.16	6.01	29	< 0.001	Significant
AA	3.09	4.12	1.03	5.76	29	< 0.001	Significant
TU	3.35	4.22	0.87	4.45	29	< 0.001	Significant
VLD	2.94	4.07	1.13	6.38	29	< 0.001	Significant

The paired t-tests reveal statistically significant post-intervention improvement in every variable. Specifically, Student Engagement increased from 3.12 to 4.26 (t = 6.72), Academic Performance increased from 68.4 to 79.2 (t = 5.89), and Creative Output increased from 2.87 to 4.03 (t = 6.01). All p-values were < 0.001, confirming the statistical significance of all the post-intervention gains.

## Correlation analysis

**Fig. 9:**
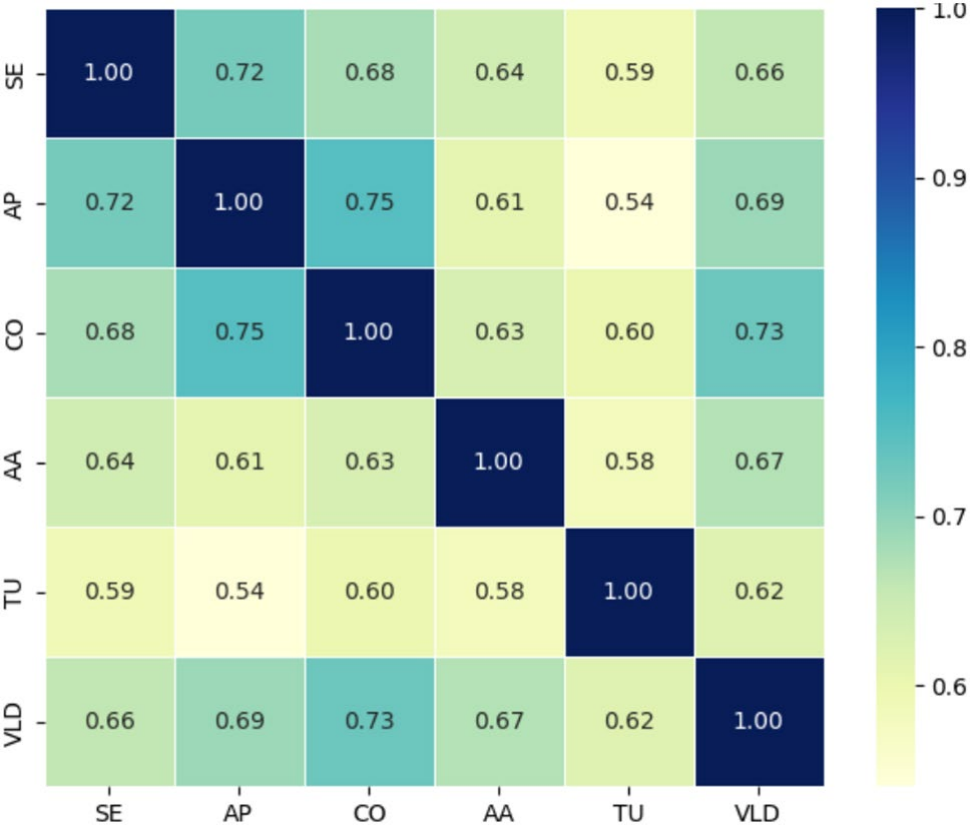
Outcome of correlation analysis.

Correlation analysis is a statistical measure used to determine the strength and direction of the relationship between two or more variables. Value can be indicated as either a directional association, suggesting that an increase or decrease in one variable will also show a similar increase or decrease in the other variable (Eq. 25). This analysis uses correlation analysis to assess the relationship between engagement, creativity, and academic performance (Fig. 9).

25 $$q= \frac{z(\sum us)-(\sum u )(\sum s ) }{\sqrt{[z\sum {u}^{2}-{\left(\sum s\right)}^{2}]}[z\sum {v}^{2}-{\left(\sum s\right)}^{2}]}$$

The correlation coefficient ($q$) is calculated using this equation: the $q$ measures the degree of linear association between two variables, such as SE ($\sum us)$ and perceived system effectiveness ($s$), again to assess the impact of AI and AR, and art education. It identifies the extent to which improvements in one variable will lead to changes in the other variable.

The correlation matrix highlights the variables and their strong positive relationships associated with the variables: SE, AP, CO, AA, TU, and VLD. Findings show that the strongest correlations were reported for AP and CO (0.75), and CO and VLD (0.73), suggesting that all three learning outcomes are mutually interconnected.

## Discussion

Integrate AR and AI in a smart classroom setting to improve student engagement, creativity, and aesthetic learning in photographic art education. Faster R-CNN^30^ barely has any comparisons when it comes to accuracy. The major letdown is the extreme inertia in computation because of its two-stage object detection. The SSD^30^ is comparatively fast but loses accuracy on small objects. YOLOv5n^30^ is extremely lightweight and really fast, though it will lose something in terms of precision when many objects are within complex scenes. All models require big data with an extensive amount of labeled data and computational resources to run. To overcome the limitations, the present research presents the comparative data table and empirical values. The proposed DRNN-based AI–AR has performed better than the other traditional models for accuracy, precision, recall, and F1-score. Statistically significant improvements were indicated, including key variables—student engagement (1.14), academic performance (10.8), and visual literacy (1.13). These findings attest to the model’s educational impact and technological advantage over previous models in smart classroom settings.

## Conclusion

The research investigated at how to incorporate AI and AR with photographic art instruction in smart classrooms to improve student engagement, creativity, academic achievement, and aesthetic appreciation. The technology coupled saliency-based image preprocessing with a TensorFlow-implemented DRNN to enable intelligent image creation and feedback. Data were acquired from both open-source Kaggle datasets (1,020 records) and a pilot study with 30 participants, with outcomes assessed using metrics for performance, surveys, and observation. The results demonstrated significant gains in educational outcomes, with the DRNN model beating previous vision-based models (F-RCNN, SSD, YOLOv5n) with 97.18% accuracy, 97.33% precision, 96.95% recall, and a 97% F1-score. Paired t-tests showed statistically significant improvements in student engagement, academic performance, creativity, visual literacy, and technological usability. The pilot research faced limitations such as a small sample size, limited discipline diversity, and potential constraints in generalizing results across educational contexts, while issues related to data privacy and ethical AI/AR use remained unresolved. Future research should explore diverse student populations, AI-AR systems across art forms and subjects, robust data security, informed consent, responsible use frameworks, and cross-disciplinary learning models to enhance AI and AR application in education.

## Data Availability

The datasets used and/or analysed during the current study are available from the corresponding author on reasonable request.
